# Supplementary material for: Transcriptomic Insights into Mechanisms of Early Seed Maturation in the Garden Pea (Pisum sativum L.)
Source: Cells. 2020 Mar 23;9(3):779. doi: 10.3390/cells9030779 (PMC7140803; doi:10.3390/cells9030779)

Sprint−2,20(2)

Global Summary

%DE = 0.24

genes with fdr < 0.2 = 6426 (2807 + / 3619 −)

genes with fdr < 0.1 = 5910 (2605 + / 3305 −)

genes with fdr < 0.05 = 5629 (2483 + / 3146 −)

genes with fdr < 0.01 = 5016 (2218 + / 2798 −)

genes in genesets = 0

<FC> = 0

<t−score> = 0

<p−value> = 0

<fdr> = 0.76

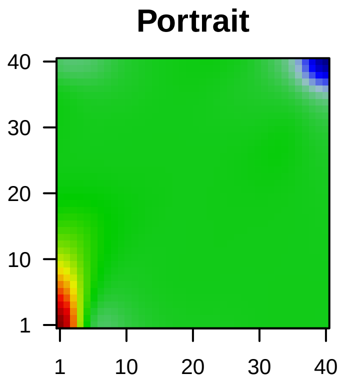

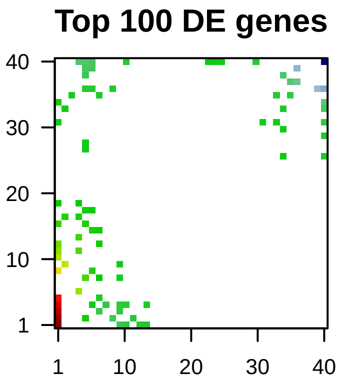

Global Genelist

Rank	ID	log(FC)	p−value / fdr	Metagene	Description
1	TRINITY_DN2	0.04	0.0000000000000002x10^-17 0.0000000000000002 (overprinted)		
2	TRINITY_DN2	−0.06	0.00000000000000002x10 0.0000000000000002 (overprinted)		
3	TRINITY_DN1	0.21	0.0000000000000002x10 0.0000000000000002 (overprinted)		
4	TRINITY_DN4	−0.04	0.0000000000000002x40 0.0000000000000002 (overprinted)		
5	TRINITY_DN4	−0.1	0.0000000000000002x4 0.0000000000000002 (overprinted)		
6	TRINITY_DN3	0.04	0.0000000000000002x30 0.0000000000000002 (overprinted)		
7	TRINITY_DN3	−0.06	0.0000000000000002x26 0.0000000000000002 (overprinted)		
8	TRINITY_DN3	−0.04	0.0000000000000002x2 0.0000000000000002 (overprinted)		
9	TRINITY_DN3	0.05	0.0000000000000002x16 0.0000000000000002 (overprinted)		
10	TRINITY_DN3	−0.03	0.0000000000000002x4 0.0000000000000002 (overprinted)		
11	TRINITY_DN3	−0.04	0.0000000000000002x28 0.0000000000000002 (overprinted)		
12	TRINITY_DN3	0.19	0.0000000000000002x12 0.0000000000000002 (overprinted)		
13	TRINITY_DN1	0.89	0.0000000000000002x9 0.0000000000000002 (overprinted)		
14	TRINITY_DN1	−0.15	0.0000000000000002x33 0.0000000000000002 (overprinted)		
15	TRINITY_DN1	−0.05	0.0000000000000002x35 0.0000000000000002 (overprinted)		
16	TRINITY_DN1	0.03	0.0000000000000002x31 0.0000000000000002 (overprinted)		
17	TRINITY_DN1	−0.06	0.0000000000000002x26 0.0000000000000002 (overprinted)		
18	TRINITY_DN1	−0.15	0.0000000000000002x39 0.0000000000000002 (overprinted)		
19	TRINITY_DN1	−0.04	0.0000000000000002x1 0.0000000000000002 (overprinted)		
20	TRINITY_DN1	−0.1	0.0000000000000002x2 0.0000000000000002 (overprinted)		

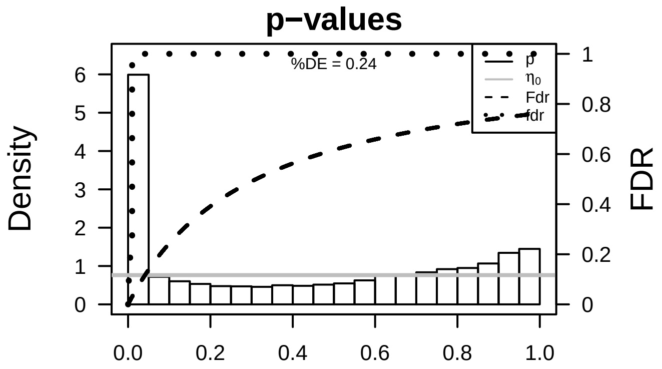

Supplement: Supplementary file 1 [file cells-09-00779-s001.zip › Supplementary materials/FileS2.zip!/FileS1/Summary Sheets - Samples/Sprint.2.20.2..pdf]
